# Apple-derived miR-482a-3p decreases c-MYB expression and proliferation of human intestinal cell lines

**DOI:** 10.1371/journal.pone.0347565

**Published:** 2026-04-28

**Authors:** Emanuela Pasculli, Marica Cariello, Maria Arconzo, Carla Gualtieri, Anca Macovei, Silvia Cultrera, Raffaella Maria Gadaleta, Elena Piccinin, Antonio Moschetta

**Affiliations:** 1 Department of Interdisciplinary Medicine, University of Bari “Aldo Moro”, Bari, Italy; 2 INBB National Institute for Biostructure and Biosystems, Rome, Italy; 3 Department of Biology and Biotechnology “L. Spallanzani”, University of Pavia, Pavia, Italy; 4 Department of Translational Biomedicine and Neuroscience (DiBraiN), University of Bari “Aldo Moro”, Bari, Italy; Columbia University Irving Medical Center, UNITED STATES OF AMERICA

## Abstract

MicroRNAs (miRNAs) are small endogenous RNAs involved in gene expression modulation at post-transcriptional level, regulating several biological processes ranging from differentiation to proliferation and preservation of cell and tissue identity. Evidence describe the ability of miRNAs to move between organisms of distinct species, a phenomenon referred to as “trans-kingdom” exchange. Notably, plant miRNAs can be transferred to other species, including humans, and potentially target and modulate genes. Here, we investigated for the first time the trans-kingdom capability of apple-derived microRNAs (miRNAs) to influence human intestinal cells (HT-29 and HCT116) *in vitro*. Our data demonstrate that synthetic apple-derived miR-482a-3p and miR-858 can be internalized by human colonic cell lines and significantly reduce cell proliferation. Through an *in silico* analysis followed by gene expression and luciferase reporter assays, we show that apple-derived miR-482a-3p targets the human c-MYB gene in intestinal cell lines, thereby directly contributing to the regulation of the enterocyte cell cycle. This study supports the trans-kingdom hypothesis by providing evidence that plant-derived miRNAs can directly modulate enterocyte lifespan and metabolic activity.

## Introduction

MicroRNAs (miRNAs) are endogenous, single-stranded RNAs with a length of 22–25 nucleotides [1]. Because of their implication in gene silencing at the post-transcriptional level, miRNAs have crucial roles in various biological functions, such as development, proliferation, differentiation, apoptosis, immune response, organ development, and maintenance of cell and tissue identity [2]. Dysregulation of miRNA expression leads to pathological conditions, including metabolic disorders, cardiovascular disease, and cancer [2]. The functional role of miRNAs in gene silencing initially arose as a mechanism targeted at viruses and transposable elements. Subsequently, the concept that miRNAs might pass between organisms of the same species and of different species emerged [3]. MiRNAs passing from a species to another leads to the so-called trans-kingdom model, a mechanism first studied between plants and their parasites and now especially between plants and humans, via food intake [3–12].

Zhang *et al.* discovered that miR-168a, a specific miRNA found in rice, was present in the serum of a small group of healthy Chinese subjects, proving the trans-kingdom cross-talk between plants and humans via food intake [13]. Futhermore, in mice fed with fresh rice, miR-168a down-regulated the Low-Density Lipoprotein Receptor Adaptor Protein 1 (*LDLRAP1*), involved in LDL removal from plasma [13]. Plant miRNAs were also found in breast milk of women that adhered to different diets, mainly composed of fruits and vegetables [14]. Interestingly, several studies demonstrated the ability of exogenous plant miRNAs to enter the human body via food intake, resist gastrointestinal conditions, and act on human gene expression [5,11,12]. Moreover, exogenous miRNAs, such as miR-159 and miR156a, found in broccoli and green veggies, respectively, have been studied for their ability to target human genes [15,16]. Specifically, miR-159a was able to down-regulate Transcription Factor 7 (*TCF7*) [14], dysregulated in breast cancer [17]. MiR-156a reduced the expression of Junctional Adhesion Molecule-A (*JAM-A)*, which plays a crucial role in the initial stages of atherosclerotic plaque formation [16,18]. In this scenario, further studies are needed to investigate the ability of plant miRNAs to regulate human genes in a trans-kingdom manner.

In the present study, using an *in vitro* system, we investigated the role of apple miRNAs in regulating human intestinal cell lines, as the intestine represents the major absorption site in the gastrointestinal tract, facilitating interactions between food and the human body. Apple is one of the most highly consumed fruits at a global level [19]. Therefore, due to its economic importance, studies dedicated to sequence and assemble its genome, are available [20]. Intriguingly, we identified two apple-derived miRNAs that are able to communicate with intestinal cells reducing their growth.

## Methods

### 1. Fruits and juice

The fruits used in the present study, namely apple (*Malus domestica*) cv. Golden delicious, pear, kiwi, and orange, were purchased from local greengrocers. Apple juice as well as juice from fruit mix, were prepared from fresh fruit without seeds and peel using an electric juice extractor without water addition. Experiments were conducted in three technical replicates and repeated three times.

### 2. Cell culture

Colorectal carcinoma cell lines HT-29 and HCT116 as well as human embryonic kidney HEK-293 cells were obtained from the American Type Culture Collection (ATCC, Manassas, VA, USA). HT-29 cell line was maintained in McCoy’s 5A (Modified) Medium (Thermo Fisher Scientific, USA), supplemented with 10% heat-inactivated Fetal Bovine Serum (FBS) and 1% penicillin-streptomycin (10,000 U/mL), and cultured in 75 cm^2^ vented cell culture flasks. HCT116 and HEK-293 lines were maintained in Dulbecco’s modified eagle medium (DMEM; Thermo Fisher Scientific, USA) supplemented with 10% heat-inactivated FBS and 1% penicillin-streptomycin (10,000 U/mL) in 75 cm^2^ vented cell culture flasks. All cell cultures were cultured at 37°C and 5% CO_2_. Colorectal carcinoma cell lines were split when reaching approximately 70% confluency, while HEK-293 were split at 90% confluency.

### 3. RNA and miRNA extraction

Total RNA was extracted using the Qiazol reagent (Qiagen) following the manufacturer’s instructions. cDNA was synthesized by retrotranscribing 1 μg of total RNA in a total volume of 50 μL using a High Capacity cDNA Reverse Transcription Kit (Thermo Fisher Scientific, USA), applying the manufacturer’s instructions. MiRNA isolation was performed using mirVana™ miRNA Isolation Kit (Thermo Fischer Scientific, USA) following the manufacturer’s guidelines. RNA quality was assessed by 260 nm absorbance with a Nanodrop spectrophotometer (NanoPhotometer N60, Implen, DE). For miRNAs expression analysis, reverse transcription was made using TaqMan microRNA Reverse Transcription Kit (Thermo Fisher Scientific, USA), following manufacturer’s protocol.

### 4. Apple miRNA and miRNA mimic transfection

HT-29 and HCT116 cell lines were split when reaching ≥80% confluency and transfected with a mix containing 25 nM of miRNAs isolated from apple or 50 nM of mirVana miRNA mimic Malus domestica (mdm)-miR858 (Thermo Fisher Scientific, USA; Cat. No. 4464066, Product ID MC25773) or with 50 nM mirVana miRNA mimic mdm-miR482a-3p (Thermo Fisher Scientific, USA; Cat. No. 4464066, Product ID MC25918), serum-free medium (Opti-MEM I Reduced-Serum Medium, Thermo Fisher Scientific, USA; Cat. No. 11058021) TransIT-X2® Dynamic Delivery System (Mirus Bio LLC, USA) following the manufacturer’s instructions. After that HT-29 were seeded into 12-well plates at a density of 0.5 x 10^5 cells/mL in a complete growth medium of 1 mL. HCT116 were seeded into 12-well at a density of 0.3 x 10^5 cells/mL in a complete growth medium of 1 mL. HT-29 were seeded into 6-well plates at a density of 1.25 x 10^5 cells/mL in a complete growth medium of 2.5 mL and HCT116 were seeded into 6-well plates at a density of 1 x 10^5 in a complete growth medium of 2.5 mL. All experimental controls were treated with an equal concentration of a non-targeting control mimic sequence (mirVana™ miRNA Mimic, Negative Control #1; Thermo Fisher Scientific, USA; Cat. No. 4464058) to evaluate non-sequence-specific effects. Total RNA and protein extract were collected 72 and 96 hours post-transfection and stored at −80°C for subsequent downstream analysis. Experiments were conducted in three technical replicates and repeated three times.

### 5. *In silico* analysis and real-time quantitative PCR

Prediction of miRNA targets was assessed by using RNAhybrid tool, using apple-derived miRNA sequences registered in miRBase database and human 3’UTRome sequence dataset. Up to 50 putative targets were taken into account as previously reported for accurate analysis [21], considering the Minimum Free Energy (MFE) value at a threshold of −34.7 kcal/mol. The gene set enrichment analysis was performed using the EnrichR package, according to Kyoto Encyclopedia of Genes and Genomes (KEGG) Pathways Database based on p–value ranking. For gene expression studies, RT-qPCR assays carried out in 96-well plates using the Master Mix Power SYBR Green (Thermo Fisher Scientific, USA) using the QuantStudio5 machine (Thermo Fisher Scientific, USA). Human TATA-box binding protein (*TBP*) was used as an internal control. Validated primer sequences used for RTqPCR are listed in Table 1.

**Table 1:** List of validated primer sequences used for RTqPCR.

Gene	Forward (5’-3’)	Reverse (5’-3’)
IL4R	CTGACCACGTCATCCATGAG	GTGGAAGATGAATGGTCCCA
c-MYB	CTATTACCACATTTCTGAAGCACAA	TAGCTCATTCTCGGTTGACATTAG
MAP4K4	GAGCACTCACACCAAAGTCA	CTGGCACATCTTCACATTCATC
CYCLIN D1	CGTGGCCTCTAAGATGAAGGA	CGGTGTAGATGCACAGCTTCT
CYCLIN E1	GACCCACAGAGACAGCTTGGA	GTTCAGACAAACATGGCTTTCTTTG
COX2	TTCCAGATCCAGAGCTCATTAAA	CCGGAGCGGGAAGAACT
c-MYC	CCACCACCAGCAGCGACT	CAGAAACAACATCGATTTCTTCCTC
TBP	CACGCAGGAAGAGGTCATCA	AGGGAAAGCTATGCCTTCAAA

RT-qPCR for miRNAs expression was performed using pre-validated TaqMan Assays and TaqMan Universal Master Mix (Thermo Fisher Scientific, USA), following the manufacturer’s instructions. Normalization was performed using plant U6 (Thermo Fisher Scientific, USA, Cat. No. 4398987, Assay ID CTMFW7W) as an internal control in miRNA extracted from apple and fruit mix pieces and juice; U6 snRNA (Thermo Fisher Scientific, USA, Cat. No. 4440887, Assay ID 001973) was used as internal control in human transfected cell lines. All reactions were run in triplicate. Relative quantification was performed using the ΔΔCT method. All experiments were conducted in three technical replicates and repeated three times.

### 6. Western blotting

HT-29 and HCT116 cells were lysed with RIPA buffer (Sigma-Aldrich, USA)) containing a protease inhibitor cocktail (Abcam, Cambridge, UK) and a phosphatase inhibitor cocktail (Abcam, Cambridge, UK). The lysates were centrifuged at 13,000 g at 4°C for 15 min and then the supernatants were used to evaluate protein concentrations using the Bradford assay (Bio-Rad Protein Assay, Hercules, CA). Equal amounts of protein samples (30 μg for HT-29 cells and 40 μg for HCT116 ones, because of different c-Myb protein levels in the considered cell lines, according to Human Protein Atlas) were separated on a 12% sodium dodecyl sulfate-polyacrylamide gel and transferred onto nitrocellulose membranes (Sigma-Aldrich). Membrane blocking was performed using 0.1% Tween 20 in Tris-buffered saline/(T-TBS) + 5% bovine serum albumin (BSA) and probed with primary antibodies against c-Myb (Rabbit Anti-Human c-MYB; Abcam, Cambridge, UK; Cat. No. ab109127), and Gapdh (Mouse Anti-Human Gapdh; Genetex; Cat. No. GT239). Membranes were washed in T-TBS and incubated with horseradish peroxidase-conjugated secondary antibodies, Goat Anti-Rabbit IgG H&L (Abcam, Cambridge, UK, Cat. No. ab97051) and Goat Anti-Mouse IgG H&L (Abcam, Cambridge, UK; Cat. No. ab6789), respectively. Membranes were developed with the Clarity Western ECL Substrate (Biorad, Cat. No. #1705060). Images were acquired using the ChemiDoc Touch Imaging System (Biorad) and quantified by ImageLab Software. Gapdh band intensity was used for equal loading control and normalization. Experiments were conducted in three technical replicates and repeated three times.

### 7. 3’-UTR luciferase reporter assay

HEK-293 cells (20000 cells/well) were seeded into 96-well plates and then transfected in antibiotic-free DMEM with 50 nM mirVana miRNA mimic mdm-miR482a-3p (Thermo Fisher Scientific, USA; Cat. No. 4464066, Product ID MC25918) or negative control (mirVana™ miRNA Mimic, Negative Control #1; Thermo Fisher Scientific, USA; Cat. No. 4464058) using TransIT-X2® Dynamic Delivery System (MirusBio LLC, USA), following manufacturer’s instructions. After 24h, 3’-UTR reporter vectors (2,5 µg/well) were transfected with Transit-X2 System (MirusBio LLC, USA) according to the manufacturer’s instructions. After an additional 24h, luciferase activity was measured using the Dual-Glo Luciferase Assay System (Promega, Madison, WI, USA). Samples were prepared and quantified following the kit datasheet. Firefly luciferase activity was normalized to the corresponding *Renilla* luciferase activity. C-MYB miRNA 3’-UTR target expression clone (Promoter: sv40; Dual reporter: hLuc-hRLuc; Cat. No. HmiT130575-MT06) and mutated c-MYB miRNA 3’-UTR target expression clone (CUS-20250902-152047482) were provided by Genecopoeia (Rockville, MD, USA). Empty 3’-UTR miRNA Target clone control vector for pEZX-FR02 (CmiT000001-MT06) was used as an internal transfection control. Experiments were performed in four technical replicates and repeated three times.

### 8. Sulphorhodamine B assay (SRB)

For SRB assay, cells (4000cells/well for HT-29 and 2000cells/well for HCT116) were grown in 96-well plates for 120h, fixed every 24 hours, from 0 to 120h, using cold 50% trichloroacetic acid (TCA) for 1h at 4°C. Subsequently, cell lines were stained using SRB (Sigma-Aldrich) 0.4% in 1% acetic acid. Tris-Base 10 mmol/L was used for solubilization and the absorbance of protein biomass, proportional to the cell number, was read at 550 nm with microplate spectrophotometer Epoch 2 (Agilent-Biotek, USA). Background absorbance was subtracted from all wells. The percentage of cell growth was calculated by normalizing treatments to T0 using the following formula: % cell growth = absorbance sample Tn/absorbance sample T0 x 100. Experiments were conducted in eight technical replicates and repeated three times.

### 9. Statistical analysis

All results are expressed as mean ± SEM in dot plots. Data distribution and gene expression statistical analysis were performed using GraphPad Prism software (v5.0; GraphPad Software Inc., San Diego, CA). Comparisons of the two groups were conducted using the Mann-Whitney U test. Comparison between three groups was performed using One-way ANOVA followed by Tukey’s test for multiple comparisons. A *p*-value of 0.05 was considered significant.

## Results

### 1. Apple highly expresses miR-482a-3p and miR-858

**Fig 1:**
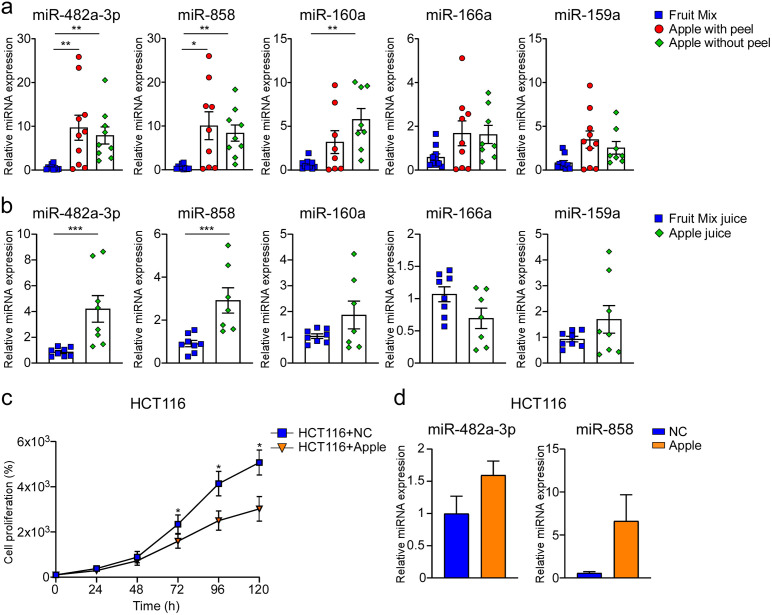
Apple highly expresses miR-482a-3p and miR-858. Expression profile of five plant miRNAs (miR-482a-3p, miR-858, miR-160a, miR-166a and miR-159a) in (a) pieces of apple, with and without peel, and fruits mix, and (b) apple and fruits mix juice. All data are expressed as mean±SEM and statistical significance was assessed by 1-way ANOVA followed by Kruskal-Wallis test (*p < 0.05; **p < 0.01) concerning miRNAs expression in apple and fruit mix pieces; Mann-Whitney U test was performed in experiments using fruit mix and apple juice (***p < 0.001). Proliferation of HCT116 cell lines with miRNAs directly extracted from apple at different time points (c). Apple-derived miRNA expression in HCT116 72-hours post-transfection (d). All data are expressed as mean±SEM and statistical significance was assessed by Mann-Whitney U test (*p < 0.05).

Literature data suggest a high abundance of five miRNAs (miR-482a-3p, miR-858, miR-160a, miR-166a and miR-159a) in fruits [21–27]. To determine the most abundant miRNAs in apples (*Malus domestica* cv. Golden delicious), we analysed the expression of these five miRNAs, using a fruit mix (pear, kiwi, orange and apple) as a calibrator. Initially, miRNA profiles were investigated in pieces of either fruit mix or apple, both with and without peel (Fig 1a). A significantly higher expression of miR-482a-3p (p = 0.002) and miR-858 (p = 0.01; p = 0.003) was found in apple with and without peel compared to the fruit mix. Also, miR-160a in apple without peel compared to fruit mix was detected (p = 0.002). No difference was observed in miR-166a and miR-159a expression in apples with or without peel compared to the fruit mix (Fig 1a). Since Liang *et al.* demonstrated that fruit miRNAs are present in juice obtained with an electric extractor [5], we also analysed the miRNAs expression in a fresh fruit juice. When juice was tested, a significant increase in miR-482a-3p (p < 0.001) and miR-858 (p < 0.001) expression in apple juice compared to fruit mix juice (Fig 1b) was observed. No significant difference between fruit and apple juice was found for the other considered miRNAs (Fig 1b). These results indicate that miR-482a-3p and miR-858 are preferentially expressed in apple fruits, both when used as pieces and juice. Therefore, we select these miRNAs for *in vitro* analysis.

In order to analyse the ability of exogenous apple miRNAs to affect human cells, HCT116 were transfected with miRNAs extracted from apple. In this cell line, we observed a significant decrease in cell proliferation 72-hours post-transfection (p = 0.031; p = 0.033; p = 0.029) (Fig 1c). Furthermore, we found an increase expression of selected miR-482a-3p and miR-858 in transfected cells confirming their role in cell proliferation reduction (Fig 1d).

### 2. Apple miR-482a-3p and miR-858 decrease proliferation of human intestinal cell lines

**Fig 2:**
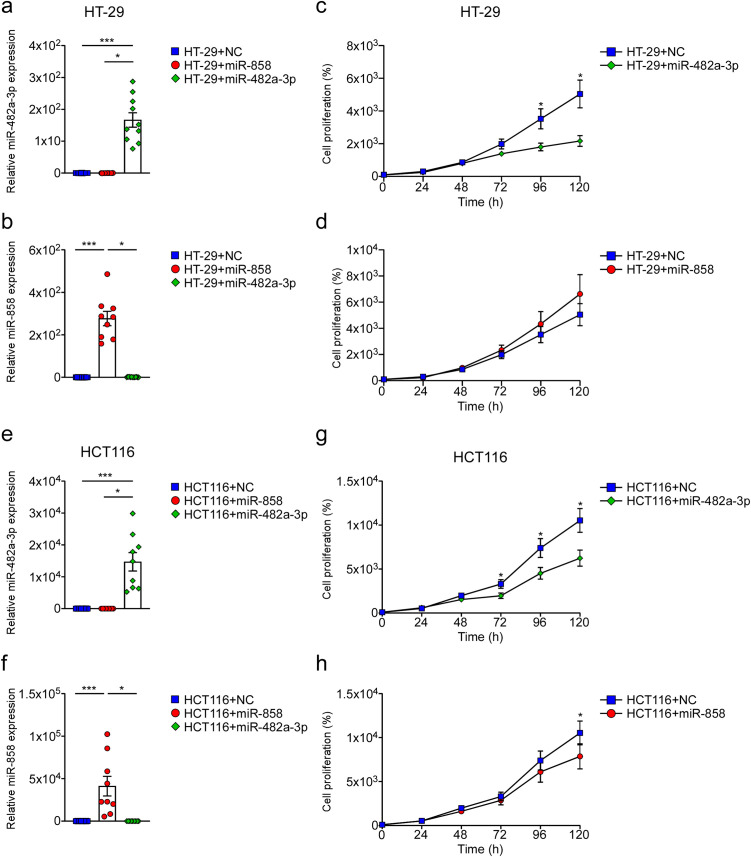
Apple miR-482a-3p and miR-858 decrease proliferation of human intestinal cell lines. miR-482a-3p and miR-858 expression in HT-29 cell lines 96-hours post-transfection (a; b). miR-482a-3p and miR-858 expression in HCT116 cell lines 72-hours post-transfection (e; f). All data are expressed as mean±SEM and statistical significance was assessed by 1-way ANOVA followed by Kruskal-Wallis test (*p < 0.05; ***p < 0.001). Proliferation of transfected HT-29 cells via SRB assay at different time points (c; d). Proliferation of transfected HCT116 cells via SRB assay at different time points (g; h). All data are expressed as mean±SEM and statistical significance was assessed by Mann-Whitney Utest (*p < 0.001).

To assess the hypothesis of plant miRNA impact on the human body, two human colorectal adenocarcinoma cell lines, HT-29 and HCT116, were transfected with miRNAs mimicking apple miR-482a-3p and miR-858 (Fig 2a, 2b, 2e, 2f). The efficiency of transfection was assessed at different time points (from 0 to 120 hours; data not shown), indicating a higher efficiency at 96 hours post-transfection in HT-29 cells (miR-482a-3p: p < 0.001; p = 0.01; miR-858: p < 0.001; p = 0.03) (Fig 2a, 2b) and after 72 hours in HCT116 cells (miR-482a-3p: p < 0.001; p = 0.03; miR-858: p < 0.001; p = 0.03) ((Fig 2e, 2f). We analysed cell proliferation after miRNA transfection via SRB assay in HT-29 and HCT116 cells. At 96 (p < 0.001) and 120 (p < 0.001) hours post-transfection we observed a significant decrease in HT-29 transfected with miR-482a-3p compared with cells transfected with negative control (NC) (Fig 2c). A mild increase in proliferation of HT-29 transfected with miR-858 was also detected (Fig 2d). In the same way, HCT116 transfected with miR-858 and miR-482a-3p exhibited a significant decrease of proliferation compared with those transfected with negative control (Fig 2g, 2h). In particular, HCT116 transfected with miR-482a-3p displayed a significant decrease in proliferation already after 72 hours post-transfection (p < 0.001) (Fig 2g), while HCT116 transfected with miR-858 showed a reduction in proliferation only at 120 hours post-transfection (p < 0.001) (Fig 2h). Overall, these data indicate the ability of miRNAs mimicking apple ones to have an effect on human cell line proliferation, leading us to better explore the mechanism behind it.

### 3. *c-MYB* expression is reduced in the HT-29 and HCT116 cell lines transfected with miR-482a-3p

**Fig 3:**
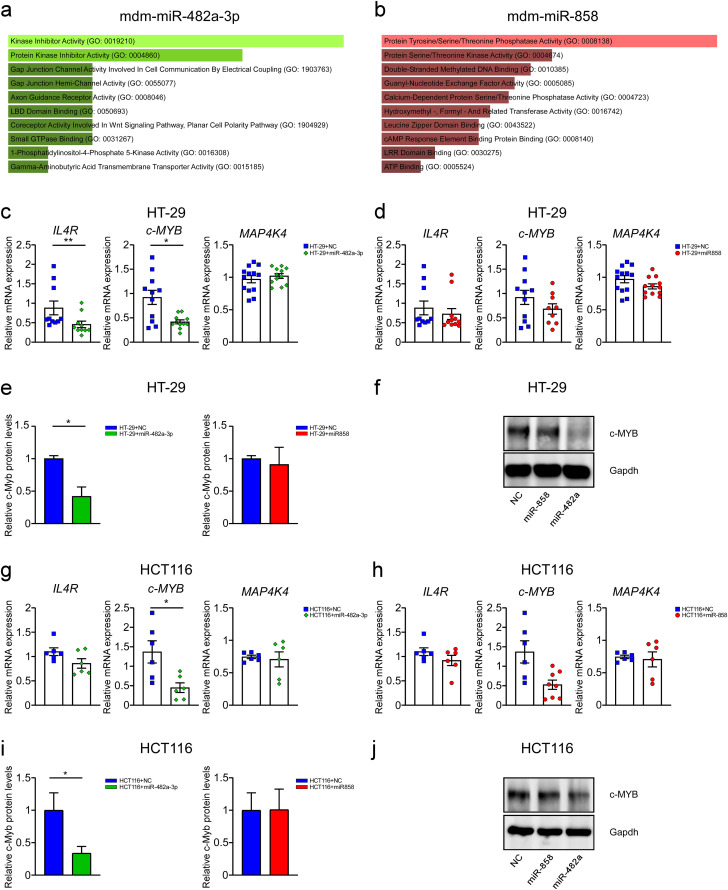
The *c-MYB* expression is reduced in the HT-29 and HCT116 lines transfected with miR-482a-3p. MicroRNA target prediction and enrichment analysis of the predicted target genes for mdm-miR-482a-3p (a) and mdm-miR-858 (b). Genes targeted by the two miRNAs were predicted using the RNAHybrid tool. The gene set enrichment analysis was performed using EnrichR. The bar graphs show the predicted human Kyoto Encyclopedia of Genes and Genomes (KEGG) pathways based on *p*–value ranking. mRNAs expression of *IL4R, c-MYB* and *MAP4K4* in HT-29 transfected cells 96-hours post-transfection (c; d). mRNAs expression of *IL4R, c-MYB* and *MAP4K4* in HCT116 transfected cells 72-hours post-transfection (g; h). c-Myb protein levels in HT-29 transfected cells 96 hours post-transfection and HCT116 transfected cells 72 hours post-transfection (e; i). Relative c-Myb protein levels were calculated using ImageLab Software (d; j). All data are expressed as mean±SEM and statistical significance was assessed by Mann-Whitney U test (*p < 0.05; **p < 0.01).

**Fig 4:**
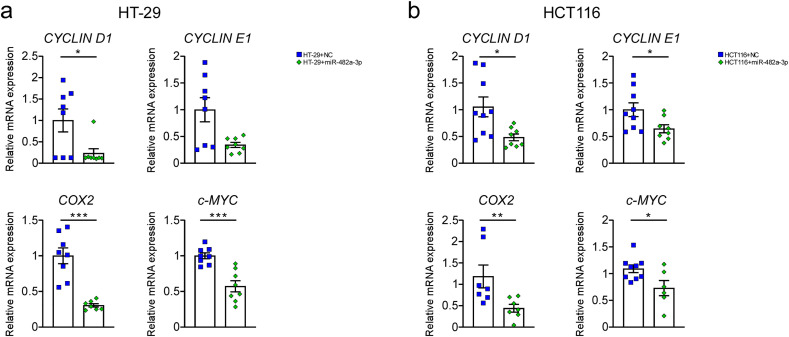
Expression of c-MYB downstream targets in HT-29 and HCT116 cells transfected with miR-482a-3p. mRNA expression of *CYCLIN D1, CYCLIN E1, c-MYC* and *COX2* in HT-29 cell lines 96-hours post-transfection (a); mRNA expression of *CYCLIN D1, CYCLIN E1, c-MYC*, and *COX2* in HCT116 cell lines 72-hours post-transfection (b). All data are expressed as mean±SEM and statistical significance was assessed by Mann-Whitney U test (*p < 0.05; **p < 0.01; ***p < 0.001).

In order to evaluate the ability of miR-482a-3p and miR-858 to target human genes, we conducted *in silico* analysis to predict putative miRNA target candidates. Aligning mdm-miR-482a-3p and mdm-miR-858 sequences from the miRBase database and the human genome deposited in NCBI as a reference, putative miRNA target candidates were predicted. Subsequently, among the different predicted targets, based on the interaction between miRNA seed region and mRNA 3’-UTR, according to the MFE, a few genes were chosen for further analysis depending on their role in cancer-related pathways. Indeed, according to the KEGG Pathway Database analysis, mdm-miR-482a-3p putative target genes are primarily involved in protein kinase inhibitor activity (Fig 3a) and mdm-miR-858 in protein tyrosine/serine/threonine kinase activity (Fig 3b). Specifically, *interleukin-4 receptor (IL4R)*, *c-MYB*, and *MAP4K4* were selected as human putative targets given to their involvement in colon cancer, according to literature data [28–30]. Indeed, we analysed *IL4R*, *c-MYB* and *MAP4K4* gene expression in HT-29 and HCT116 cells transfected with miR-482a-3p and miR-858 (Fig 3c, 3d, 3g, 3h). HT-29 cells transfected with miR-482a-3p showed a significant decrease in *IL4R* (p = 0.007) and *c-MYB* (p = 0.02) gene expression after 96 hours post-transfection compared to HT-29 transfected with negative control (Fig 3c). In HT-29 cells transfected with miR-858 we did not observe significant differences in the expression of these genes (Fig 3d). Furthermore, we found a significant reduction of c-Myb protein expression (p = 0.04) in HT-29 transfected with miR-482a-3p (Fig 3e, 3f). No differences were found in HT-29 transfected with miR-858 (Fig 3d, 3f). To further confirm our results, we investigate the gene expression in HCT116 transfected cell lines, showing that miR-482a-3p significantly downregulated *c-MYB* gene expression (p = 0.003) 72 hours post-transfection compared with negative control (Fig 3g). No difference in *c-MYB* expression was observed in HCT116 transfected with miR-858 (Fig 3h). In HCT116 cells transfected with both miR-858 and miR-482a-3p we did not observe significant differences in *IL4R* and *MAP4K4* mRNA expression (Fig 3g, 3h). In line with our results, HCT116 transfected with miR-482a-3p presented a reduction of c-Myb protein expression (p = 0.03) compared to controls (Fig 3i, 3j). Overall, these data demonstrate the ability of miR-482a-3p to reduce *c-MYB* expression thus inhibiting human colorectal adenocarcinoma cell growth. Moreover, to prove the ability of apple-derived miR-482a-3p to affect *c-MYB* expression, we analysed *c-MYB* downstream target expression *CYCLIN D1, CYCLIN E1, COX2* and *c-MYC* [31–34]. HT-29 and HCT116 cells transfected with miR-482a-3p showed a significant reduction in *CYCLIN D1* (HT-29: p = 0.03; HCT116: p = 0.02), *COX2* (HT-29: p < 0.001; HCT116: p = 0.007), and *c-MYC* (HT-29: p < 0.001; HCT116:p = 0.05) expression 96 hours and 72 hours post-transfection, respectively (Fig 4a, 4b).

### 4. Identification of human c-MYB as putative apple miR-482a-3p target

**Fig 5:**
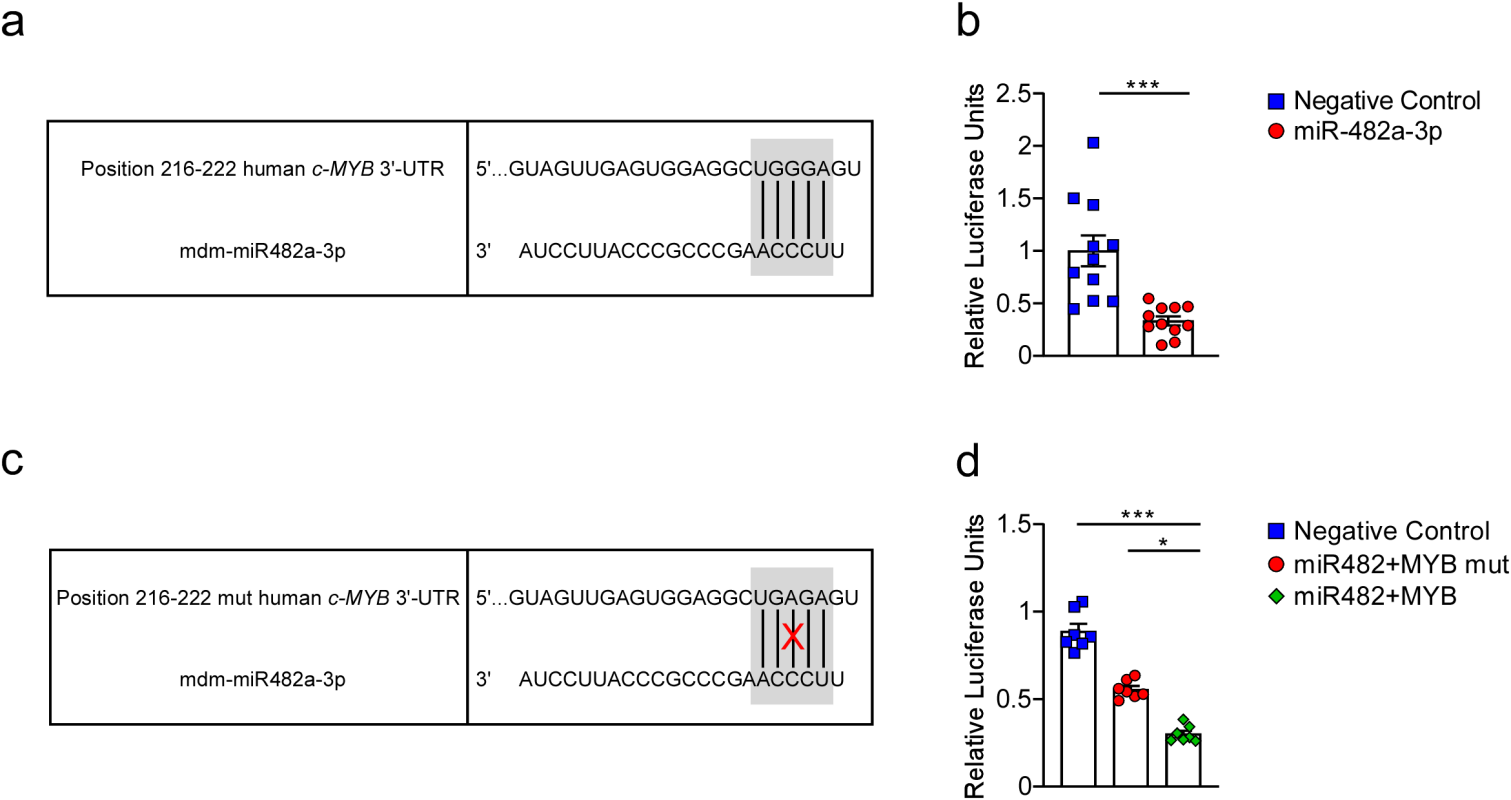
Identification of human c-MYB as putative apple miR-482a-3p target. In HEK-293 cells, miR-482a-3p mimic and negative control were transfected together with a c-MYB 3’UTR reporter construct carrying miR-482a-3p binding sequence (a); changes in luciferase activity confirm the ability of miR-482a-3p to modulate c-MYB (b). All data are expressed as mean±SEM and statistical significance was assessed by Mann-Whitney U test (***p < 0.001). A seed region mutated c-MYB 3’UTR reporter construct (c) has been used to transfect HEK293 together with miR482a-3p and c-MYB 3’UTR reporter construct (d). All data are expressed as mean±SEM and statistical significance was assessed by 1-way ANOVA followed by Kruskal-Wallis test (*p < 0.05; ***p < 0.001).

To further evaluate the ability of apple miR-482a-3p to bind and downregulate human *c-MYB* mRNA, first we carried out an *in silico* bioinformatic analysis to identify the link between the seed region of mdm-miR482a-3p and the 3’-UTR of human *c-MYB* (Fig 5a). This result was used to perform a luciferase reporter assay on HEK293 cell lines. Cells were transfected with a non-targeting control mimic sequence or miRNA mimicking apple miR-482a-3p and, after 24 hours, with *c-MYB* 3’-UTR target clone vector. After an additional 24 hours, luciferase assay was accomplished and firefly and *Renilla* luciferase luminescence were measured. A 70% reduction in luciferase activity was observed in the HEK293 transfected cells with both miR-482a-3p and *c-MYB* 3’-UTR (p < 0.001), showing the ability of this exogenous miRNA to affect human *c-MYB* expression (Fig 5b). In order to evaluate the interaction between apple miR-482a-3p and c-MYB 3’UTR in the seed region, a seed-mutated 3’-UTR target clone vector has been produced (Fig 5c) and used to transfect HEK293, together with mdm-miR-482a-3p. Our results showed no significant reduction in luciferase activity in cells transfected with miRNA and c-MYB mutated 3’-UTR (miR482 + MYBmut) compared with negative control. A significant difference has been observed between HEK293 transfected with apple-derived miRNA and c-MYB (miR482 + MYB) compared to negative control (p < 0.001) and miR482 + MYBmut (p = 0.05) (Fig 5d).

## Discussion

A healthy and well-balanced diet is recognized as a crucial and modifiable factor in preventing the onset of chronic diseases, including cardiovascular and metabolic diseases as well as cancer [35]. Central to this diet are fruits and vegetables, which are abundant sources of bioactive compounds, such as polyphenols, isothiocyanates, and carotenoids, involved in the regulation of inflammatory pathways, oxidative stress and lipid metabolism [36].

In recent years, plant-derived miRNAs become increasingly recognized for their potential to exert effects on the human body via food intake [5,11–16]. The trans-kingdom hypothesis was initially conceived by Zhang and colleagues through their study on the abundance of rice miR-168a in the serum of a healthy Chinese population. Moreover, mice fed a diet enriched in rice showed a significant increase of exogenous miR-168a in serum and also in the liver, where they found that plant miRNA was able to reduce *LDLRAP1* expression. The same result has been described in HepG2 cell lines transfected with a synthetic miR-168a, further proving the ability of exogenous plant miRNA to affect human gene expression [13]. Afterwards, other research groups provided further evidence about this intriguing interplay between food-based molecules and the human body [5,11,12,14–16]. Furthermore, in the context of cancer research, several *in vitro* and *in vivo* studies have shown the interaction between exogenous miRNAs and human target genes involved in tumor onset and progression [4,6,9,15,36]. In particular, plant miR-159 abundance in serum of healthy women and patients with breast cancer showed an inverse correlation with breast cancer incidence and progression by down-regulation of *TCF7* [15]. In addition, analysis on *TCF7* expression in human colorectal carcinoma cell lines, transfected with miR159a extracted from soybean seeds, showed a reduction in cell proliferation [36].

In our study, we have shown that synthtetic miRNAs mimicking apple-derived ones, namely miR-482a-3p and miR-858, are able to reduce colorectal cancer cell lines HT-29 and HCT116 growth by modulating human *c-MYB* expression. These miRNAs were selected based on existing literature, which indicated their shared sequences among various plant species, including the model plant *Arabidopsis thaliana* [22,21], and *Malus domestica* [23,24–27]. Notably, miR-482a-3p and miR-858 have a key role in apple development, metabolism, and responses to biotic and abiotic stress, and these are highly abundant in both peel and pulp [25,26,37]. Our results support the predominant expression of miR-482a-3p and miR-858 in apple fruits compared to a fruit mix control, in form of pieces as well as juice (Fig 1a; 1b).

Previous studies claimed the efficacy of apple polyphenolic compounds in modulating the proliferation of human colon cancer cell lines, like HT-29 [38–40] and HCT116 [41], suggesting a potential role of this fruit in colon cancer prevention [42]. To evaluate if the presence of miRNAs may contribute to the anticancer role of this fruit, apple-extracted miRNAs have been transfected to HCT116 cell lines, inducing a significant reduction in cell proliferation 72-hours after transfection (Fig 1c). Starting with this result, synthetic miRNAs mimicking apple miR-482a-3p and miR-858 were used to transfect HT-29 and HCT116 cell lines (Fig 2a; 2b; 2e; 2f). Notably, apple miR-482a-3p effectively inhibited cell proliferation in both cell lines (Fig 2c; 2g), underscoring the influence of apple miRNAs on cell growth.

**Fig 6:**
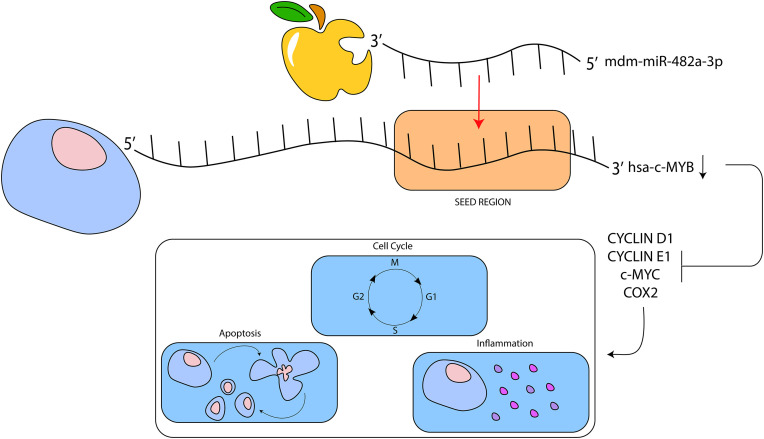
Apple-derived miR-482a-3p effect on *c-MYB* downregulation. The figure shows the interaction between apple-derived miR-482a-3p and human gene *c-MYB*. Downregulation of gene expression promotes blocking of its downstream targets, involved in some biological functions, such as cell proliferation, apoptosis and inflammation.

Given that miRNAs act through sequence complementarity [1], a bioinformatic tool was used to predict putative target genes in humans for apple miR-482a-3p and miR-858. A substantial portion of these predicted target genes were associated with pathways linked to cancer development and progression (Fig 3a; 3b). Among the genes implicated in colon cancer progression, Interleukin-4 Receptor (*IL4R*), *c-MYB* and *MAP4K4* were identified as putative target genes. In particular, *c-MYB* is an oncogenic transcription factor involved in cell proliferation, differentiation and apoptosis and it is frequently overexpressed in different tumors, including leukemia, breast cancer and colorectal cancer [29]. The mRNA expression levels of these three genes were evaluated after transfection with the selected miRNAs, observing a significant reduction in *c-MYB* mRNA and protein level (Fig 3) in HT-29 and HCT116 cells transfected with synthetic miR-482a-3p, thus suggesting the effect of this miRNA in silencing human *c-MYB*, also confirmed by the reduction in its downstream targets *CYCLIN D1, CYCLIN E1, COX2 and c-MYC* expression (Fig 4a; 4b). These data demonstrate the interaction between apple-derived miR-482a-3p and human *c-MYB* leading to dowregulation of gene involved in cell proliferation, apoptosis and inflammation (Fig 6). Further studies are needed to better clarify the involvement of trans-kingdom in human physiological and pathological gene modulation.

Taken together, our results provided a novel piece to the intricate puzzle of the trans-kingdom hypothesis supporting the role of exogenous miRNAs, mimicking miRNA-derived from ingested plants to direct human gene expression and affect human cellular mechanisms. Highlighting the significant impact of miR-482a-3p mimicking apple one on colon cancer cell proliferation via *c-MYB* targeting, we propose that plant-derived miRNAs, possibly synergistically with bioactive compounds, can harbor significant therapeutic promise, even if more studies are needed to determine whether the microRNAs concentrations found in foods are sufficient to affect cellular and physiological processes in human organism. Moreover, also plant-derived nano and microvesicles (PDVs) are rich in miRNAs and it has been reported that even these vesicles have a role in the cross-kingdom, with beneficial effect on human body [43]. This information sheds light on a compelling avenue for advancing human health, notably concerning chronic disease prevention, including potential cancer mitigation.

## Supporting information

S1 File Gels and blots images. Full gel images for HT-29 and HCT116 transfected cells (a; d). c-Myb and Gapdh cropped blot images in HT-29 transfected cells (b; c). c-Myb and Gapdh cropped blot images in HCT116 transfected cells (e; f). The loading condition legend is the following: M = Marker, C = control cells (Jurkat cell lines), 1 = cell lines transfected with Negative Control (NC), 2 = cell lines transfected with miR-858, 3 = cell lines transfected with miR-482a-3p. Images were acquired using the ChemiDoc Touch Imaging System (Biorad) and quantified by ImageLab Software. Gapdh band intensity was used for equal loading control and normalization. c-Myb and Gapdh cropped blot images in HT-29 and HCT116 have been used to generate Figure 3 panel f and j respecitvely.
(PDF)
