# Supplementary material for: Apple-derived miR-482a-3p decreases c-MYB expression and proliferation of human intestinal cell lines
Source: PLoS One. 2026 Apr 28;21(4):e0347565. doi: 10.1371/journal.pone.0347565 (PMC13123949; doi:10.1371/journal.pone.0347565)
Supplement: S1 File — Full gel images for HT-29 and HCT116 transfected cells (a; d). c-Myb and Gapdh cropped blot images in HT-29 transfected cells (b; c). c-Myb and Gapdh cropped blot images in HCT116 transfected cells (e; f). The loading condition legend is the following: M = Marker, C = control cells (Jurkat cell lines), 1 = cell lines transfected with Negative Control (NC), 2 = cell lines transfected with miR-858, 3 = cell lines transfected with miR-482a-3p. Images were acquired using the ChemiDoc Touch Imaging System (Biorad) and quantified by ImageLab Software. Gapdh band intensity was used for equal loading control and normalization. c-Myb and Gapdh cropped blot images in HT-29 and HCT116 have been used to generate Figure 3 panel f and j respecitvely. (PDF) [file pone.0347565.s001.pdf]

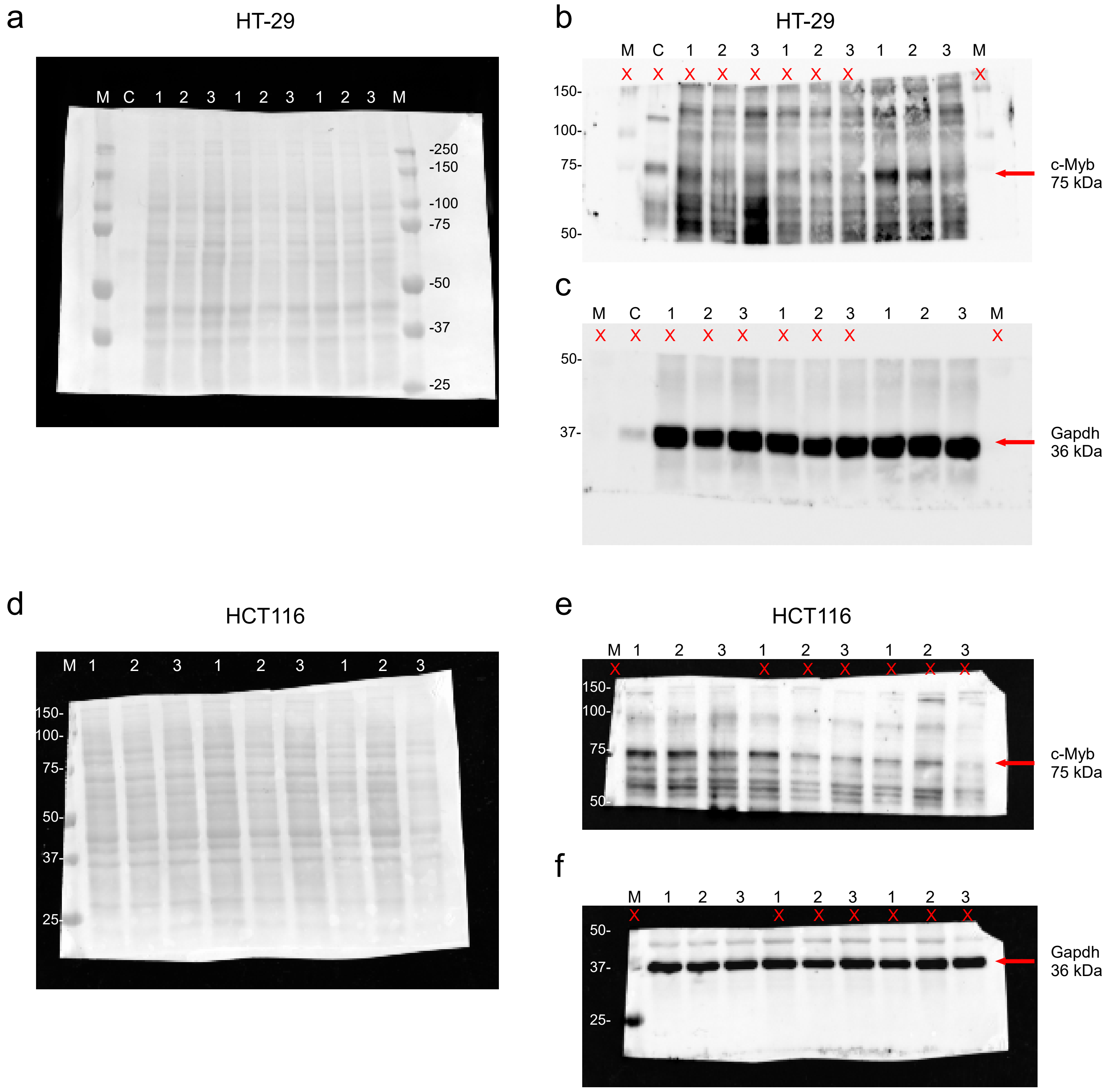

S1. Gels and Blots Images. Full gel images for HT-29 and HCT116 transfected cells (a; d). c-Myb and Gapdh cropped blot images in HT-29 transfected cells (b; c). c-Myb and Gapdh cropped blot images in HCT116 transfected cells (e; f). The loading condition legend is the following: M = Marker, C = control cells (Jurkat cell lines), 1 = cell lines transfected with Negative Control (NC), 2 = cell lines transfected with miR-858, 3 = cell lines transfected with miR-482a-3p. Images were acquired using the ChemiDoc Touch Imaging System (Biorad) and quantified by ImageLab Software. Gapdh band intensity was used for equal loading control and normalization. c-Myb and Gapdh cropped blot images in HT-29 and HCT116 have been used to generate Figure 3 panel f and j respecitvely.
